# Cerebral Cavernous Malformation: Immune and Inflammatory Perspectives

**DOI:** 10.3389/fimmu.2022.922281

**Published:** 2022-06-30

**Authors:** Tianqi Tu, Zhenghong Peng, Jian Ren, Hongqi Zhang

**Affiliations:** ^1^ Department of Neurosurgery, Xuanwu Hospital, Capital Medical University, Beijing, China; ^2^ China International Neuroscience Institute (China-INI), Xuanwu Hospital, Capital Medical University, Beijing, China; ^3^ Health Management Department, The Affiliated Hospital of Southwest Medical University, Luzhou, China

**Keywords:** cerebral cavernous malformations, vascular anomalies, endothelial cell homeostasis, inflammation, immune microenvironment

## Abstract

Cerebral cavernous malformation (CCM) is a type of vascular anomaly that arises due to the dyshomeostasis of brain capillary networks. In the past two decades, many advances have been made in this research field. Notably, as a more reasonable current view, the CCM lesions should be attributed to the results of a great number of additional events related to the homeostasis disorder of the endothelial cell. Indeed, one of the most fascinating concerns in the research field is the inflammatory perturbation in the immune microenvironment, which would affect the disease progression as well as the patients’ outcomes. In this work, we focused on this topic, and underlined the immune-related factors’ contribution to the CCM pathologic progression.

## General Description of Cerebral Cavernous Malformation (CCM)

Cerebral cavernous malformation (CCM) is a type of vascular anomaly that arises due to the defects of brain capillary networks in the central nervous system (CNS). Familial or sporadic gene mutations are widely accepted as the molecular basis of CCM (1–3). Tightly packed by the fragile vessel walls, these lesions exist dynamically. The weakness of tight junction connection and the endothelial proliferation are considered to be the main pathologies (4–6). As a consequence, flowing blood is prone to leak into the surrounding tissue through these abnormal structures. Rupture and acute bleeding can be more serious results combined with a range of complications (7–10). Evidence has described that familial and sporadic CCM patients suffered annual rupture risks of 4.3-6.5% and 0.4-3.1%, respectively, which occurred in the cerebral parenchyma, although rarely resulted in subarachnoid hemorrhage (11). Meanwhile, hemorrhagic stroke is the primary cause of disability in patients. All CCMs are thought to harbor occult bleeding, as MRI revealed that CCMs were often accompanied by hemosiderin halo signals (11–14). Moreover, although different clinical studies documented various annual symptomatic hemorrhage rates, natural history studies have identified that, both in familial and sporadic patients, past hemorrhage history is a significant risk factor for rebleeding and subsequent poor sequalae (12–16).

Although it is unlikely to become impossibly huge in the short-term, like tumors, this benign vascular disease could cause a series of neurological symptoms, including seizures, which is one of the most common reasons for hospital visit, non-specific headaches, and even neurological sensory and motor function deficits (17, 18). Once these symptoms appear, the life quality of patients can be greatly affected (7, 9, 15, 18).

For decades, progressive understanding of this vascular anomaly’s pathogenesis has been reported from clinical and basic research (19). Notably, the current view of the CCMs should be regarded as the results of the intersection of numerous factors, such as immune, hemodynamics, and angiogenesis, which formed a complex network of the disease pathology (20). Thus, we reviewed the major breakthroughs in this field in recent years and focused on the potential mechanisms between immune and the whole disease process, involving occurrence, progression, and complications, aiming to provide promising ideas for the exploration of clinical trials and therapeutic targets.

## Genetic Mutations Are the Molecular Basis

To date, it is widely accepted that the initial triggers of both familial and sporadic CCM formation have been attributed to genetic mutations. Benefited from advances in multidisciplinary development and technological innovation, mutated genes can be more comprehensively supplemented. In line with this, the signaling pathways’ regulation from the molecular level could also be summarized in a more detailed state.

### Genetic Mutations and CCM Pathogenesis

CCM diseases can be broadly divided into sporadic and familial diseases. Sporadic CCMs, which accounted for the majority (80~ 85%), usually presented as isolated lesions. Familial CCMs (15~20%) were followed by an autosomal dominant inheritance pattern and usually presented with multiple lesions (1, 19, 21, 22). The loss of function (LOF) mutations of *CCM1* (*KRIT1*, Krev interaction trapped protein 1) (23–27), *CCM2* (*MGC6407*, encoding a protein named malcavernin) (28–33), and *CCM3* (*PDCD10*, Programmed cell death protein 10) (34–36) have been thought to be the culprits of familial CCM. Once the LOF germline mutation occurs and leads to endothelial cell dysfunction, it may cause the occurrence of CCMs. Recently, the ‘two-hit’ hypothesis in CCM pathophysiology has been strongly suggested to explain some clinical phenomena. Accordingly, the ‘first hit’ could be conceptualized as germline or somatic mutation causing one allele loss in all cells. And the ‘second hit’ could be the other allele’s somatic mutation occurring in just some cells, which can happen at an indeterminate point and trigger the initiation of the lesions. However, accumulated evidence in animal models clearly demonstrated that the homozygous deletion of a CCM gene is not sufficient to cause CCM disease, leading us to think that additional determinants are required for the lesion occurrence, including immunologic and inflammatory events.

In one of our recent works, we first revealed the novel somatic activation mutations, also called gain of function (GOF) mutation, in the *MAP3K3* and *PIK3CA* genes in most sporadic CMs (cavernous malformations) (1). Through whole-exon sequencing (WES) and ddPCR (droplet digital PCR) detection, we identified a total of 73 sporadic patients with somatic missense variants in *MAP3K3* (NM_002401.3, c.1323C>G, p.Ile441Met) and *PIK3CA* (NM_006218.2, in exon 7:c.1258T>C,p.Cys420Arg; in exon 9: c.1624G>A, p.Glu542Lys, c.1633G>A, p.Glu545Lys; and in exon 20:c.3140A>Gp.His1047Arg) in our 81 patients’ cohort(90.1%). Interestingly, superposition mutations of both *MAP3K3* and *PIK3CA* were also found in 14 (19.2%) of the 73 sporadic patients. These results have also been demonstrated in recently published studies (21, 37), which undoubtedly opened a new chapter for the CCM research field. However, considering the nature of sporadic CM single lesions and its non-genetic tendency, and the low mutation detection rate in previous studies, the existence of other undiscovered somatic mutations cannot be ruled out.

### Molecular Pathology and Signaling Pathway Mechanism

**Figure 1:**
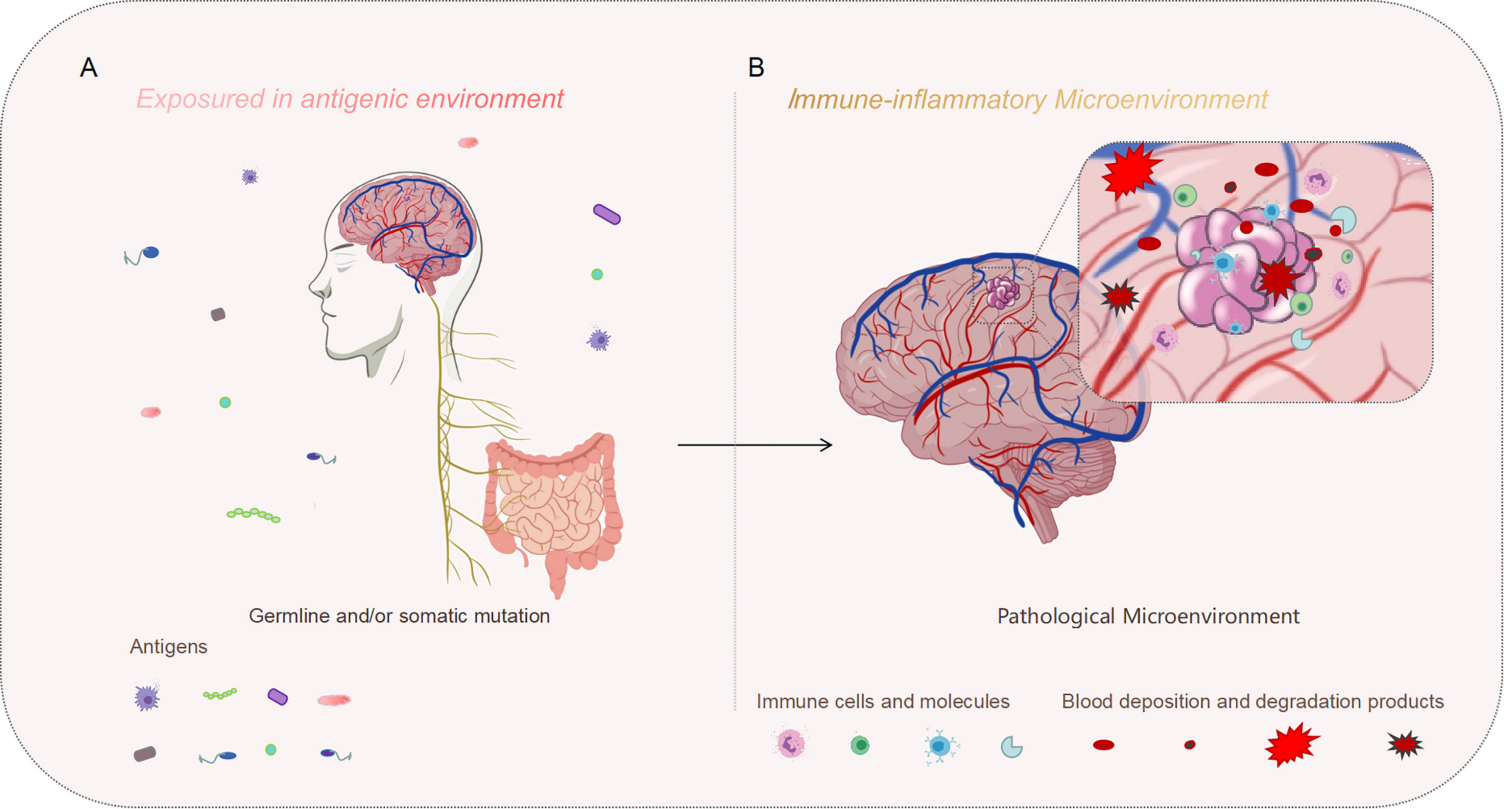
Genetic mutation might be the initial motivation, and the inflammatory perturbation in the immune microenvironment may drive the progression of the disease. **(A)** On the macro level, our bodies are constantly exposed to an antigenic environment, which could be one of the synergistic agents leading to CCM formation. **(B)** On the micro level, CCM lesions exist in the immune microenvironment. The immune microenvironment characterized by inflammation may aggravate the development of the typical pathology, meanwhile, repeated microbleeds could also exacerbate the inflammatory infiltration of this pathological background.

In general, the pathological features would reveal the essence of disease, including abnormality of endothelial cell tight junctions, proliferation of glia and endothelial cells, and even the alteration in the cytoskeleton and cell volume (4, 22, 35). Typically, CCM lesions are mulberula-like clusters of enlarged endovascular lumens. Surrounded by segmental layered basal membrane and gliotic tissue, there is little or no infiltration into the brain parenchyma (**Figure 1**). An impaired endothelial barrier would facilitate the infiltration of immune cells, which would potentially accelerate the lesion’s progression by further stimulating angiogenesis and gliogenesis (20).

Molecular signaling pathways might be the focus of non-surgical management studies, because once the pharmacological interventions work, they may be tied to these molecular targets (19, 38). Thorough summaries about signaling pathways have been made in recent works, and the classic pathways and various upstream inputs and downstream effectors implicated in CCM signaling have also been involved (19). This shall not be repeated in too much detail in this work, but several points should be noted. Firstly, since the signaling pathways converged in a large network, changes in any one molecule can affect the entire architecture. Thus, a better understanding of CCM pathomechanism would be based on a comprehensive perspective, instead of the one - sided tip. Secondly, distinct mutation-related molecular pathways may feature differently in the pathological process (19). Generally, MEKK3 (mitogen-activated protein kinase kinase kinase 3) activation might be one of the core targets, which could help to explain why both the LOF mutation in CCM genes and the GOF mutation in *MAP3K3* are sufficient to initiate CCM formation (32, 39–41).

However, unlike CCM complex or *MAP3K3* mutation, the *PIK3CA* mutation has been shown to be associated with the proliferation of lesions. *PIK3CA* mutation was previously reported for venous and lymphatic malformations (42, 43), and *PI3K/Akt/mTOR* pathway is a critical downstream target which exacerbates the lesion growth (4, 44). Strong synergy was observed when both CCM complex LOF mutations and *PIK3CA* GOF mutations co-existed (4). Thirdly, inflammatory signal is one of the major inputs in endothelial cells that affected CCM disease pathogenesis. As TLR4 (toll-like receptor 4) receptors and downstream *MEKK3-KLF2/4* signaling activated, the growth of CCM lesions in the brain were observed to accelerate (45). These unexpected findings have reminded us that non-genetic factors involved in the immune responses and inflammatory contexts could also play important roles in regulating these molecular networks (45, 46).

### Correlations Between Genotypes and Phenotypes

Different genotypes of mutations may be important factors leading to different clinical phenotypes. In familial CCMs, different mutated genes are thought to be associated with several clinical features. It was reported that there is a certain correlation between *KRIT1* mutation and cutaneous venous malformations (47–50). Individuals with *CCM2* mutations were more likely to be asymptomatic and have fewer lesions (51–53). Meanwhile, due to the significant roles of *PDCD10* in the maintenance of homeostasis of a wide range of cells, the loss of gut epithelial *PDCD10* may result in disruption of the colonic mucosal barrier, thus these individuals may be more aggressively affected by CCM diseases (54–57). In the somatic mutations, different genotypes or loci may also be associated with specific clinical phenotypes. For example, our preliminary results suggested that *PIK3CA* mutations may be associated with a higher risk of significant bleeding. Although the exact differences are not yet known, the revealing of the relationship between different mutation genotypes and clinical manifestations is an extremely interesting study, which may even guide the search for different therapeutic targets.

Strikingly, although advanced insights into the innovative knowledge of the CCM genes’ pathophysiological functions are emerging, the highly unpredictable clinical behaviors in individual patients still present a major challenge in clinical and basic research. Accumulated evidence clearly illustrated that, at least in a few cases, gene mutations alone are not sufficient to trigger CCM onset and progression (58–60). Moreover, it is also important that the highly variable severity of clinical symptoms is common, even among familial patients who carried the same genetic mutation. It has been found that in monozygotic twins who suffered the same germline mutation, exact similarity of the disease onset and clinical severity could be observed, in contrast to the different clinical severity of non-twin siblings in wide intrafamily (61). Notably, sometimes many suffered individuals could be clinically asymptomatic for a long time or even for their entire life. Once symptoms appear, there could be disastrous consequences, and the healthy quality of life could be seriously affected. Thus, what the initiator from the existence of lesions to the appearance of clinical symptoms is, and what causes the same initial encounter but different clinical outcomes, are interesting issues that we are trying to solve. The perspectives from the immune and inflammatory events described in the next part may point out a new direction for our thinking.

## Immune and Inflammatory Mechanisms for CCM Formation

The CCM lesions should be regarded as the results of a great number of additional events. Indeed, inflammatory perturbations associated with the immune microenvironment might be one of the most fascinating concerns (62). Recent study results allowed the identification of major consequences and underlined the immune-related factors’ contribution to the CCM pathologic progression. In this part, we mainly followed these insights to draw the outline of the relationship between the CCMs and related immune and inflammatory events.

### The Influence of Microbiome Is an Interesting Phenomenon

The microbiome has become a hot research topic in recent years, and has been identified to be associated with many diseases. However, the potential mechanisms through which it contributes to disease pathogenesis are still unclear. The works of Kahn et al. are particularly interesting and significant (45, 63). Firstly, the resistance to CCM formation was observed accidentally in mouse model of C57BL/6J background, which was previously identified as having 100% CCM penetrance. Then they demonstrated that, to stimulate the CCM formation, the bacterial microbiome is one of the primary sources. Meanwhile, they also revealed that, even the qualitative differences in the gut microbiome are small, they could have marked effects on the process of CCM development in animal models (45). Furthermore, the key role of TLR4in regulating inflammatory pathways in the immune microenvironment was highlighted. As their results suggested, the CCM formation would be driven by the activation of TLR4 receptors in endothelial cells, and the gut bacterial microbiome might be the leading actor (45). Given that the gut-brain axis might be a potential target, therapeutic exploration is being attempted. Obviously, the CCM formation could be inhibited in mice group which received pharmacological TLR4 blocking intervention. Similarly, the susceptibility could also be attenuated in germ-free mice, as well as in antibiotic-treated mice (63). In line with this, factors that affected the gut epithelial barrier homeostasis, involving chemical and genetic disruptions, and even colonic mucus alterations, can precipitate CCM development. As they revealed, genetic depletion of *PDCD10* could impair gut barrier homeostasis, then accelerating the CCM formation (63). The interesting results of this study are fascinating, inspiring further studies to be continued (64, 65). In this light, it is reasonable to think that the effects of individual microbiomes may help to explain clinical variability. To test this hypothesis and determine whether the manipulation of gut microbiome-host interactions is a viable therapeutic strategy, future studies are urgently needed. In addition, it is necessary to reconsider whether strategies such as protection of gastrointestinal mucosal and prevention of gastrointestinal infection should be included in the clinical management of CCM patients.

### Cumulative Direct Evidence Established the Role of Immunization

To identify what immune-related genetic modifiers would be associated with disease progression and severity, excellent research has been performed in recent decades. The Awad’s Team made an outstanding contribution to this field. Firstly, the exploration of the relationship between different gene expressions and clinical manifestations laid the basis for the gene spectrum of CCM research, which was also the origin of the discovery of immune-related molecules (52, 66, 67). Then the concepts and inflammation hypothesis in the pathogenesis of CCMs were first proposed, with laboratory investigation results (58, 68). By assessing the infiltrated immune cells and the immunoglobulin isotypes predominant in CCM lesions, they systematically described the immune responses in human CCMs. In addition, the evidence of co-localized IgG and complement membrane attack complexes in CCM lesions were reported (69). Then, the *in-situ* B-cell clonal expansion was identified, indicating antigenic stimulation in CCMs (69). Given that plasma cell-mediated immunity might be one contributory factor to CCM lesion, the evaluation of the effects of the immune response inhibition on the CCM lesions’ formation and maturation were done (70). Evidence suggested that B-cell depletion would have therapeutic benefits for CCM disease. Meanwhile, these findings also remind us that these biologics could be added into the list as potential therapeutic agents (70).

Recent results take this research to a new level (71). By using laser capture micro-dissection, plasma cells from CCM lesions were obtained, and mAbs (monoclonal antibody) were subsequently generated. Then antigens targeted by resident plasma cells in CCMs were characterized *via* detailed experiments and analysis (71). Despite the limitation of small sample size and the uncertainty of whether the mAbs would react with other antibodies, these data have given us a novel insight into the immunopathogenesis of CCM. In another piece of research, the infiltration of different subsets of leukocytes in CCM was revealed (72). Through RNA-seq analysis, increased inflammation-related genes were observed in the endothelial cells from CCM mouse model (72). Notably, the cytokines which perform the roles of inflammatory and immune cells’ recruitment, involving proinflammatory cytokines, chemokines, and adhesion molecules, are closely related to the differentially expressed genes. More importantly, neutrophils were found to have infiltrated into the CCM lesions, in the way of neutrophil extracellular traps formation (72). These findings were also validated in clinical specimens, which undoubtedly opened up new research ideas for researchers in this study field.

Advances in sequencing techniques and multi-omics analysis made it possible to access a more comprehensive understanding of the genetics and modification molecules of CCM. By using gene microarray technology, 78 abnormal expression genes related to inflammatory response in CCM lesions were observed, including 13 overexpressed immunoglobulin genes (66). Compared with control samples, probe sets revealed an up-regulation of 10 Ig genes and a distinct allele of the major histocompatibility complex in CCM samples, providing potential evidence for a characterized immune background in the CCM lesions (66). Furthermore, polymorphisms in genes of the immune-inflammatory response were also proven to be correlated with the severity of CCM disease (59, 73). Though further studies in larger sample sizes are needed, these results primarily suggested that immune-related factors might be potential predictors for phenotype variability and disease severity.

The identification of the accumulated immunologic cells could also contribute to the direct evidence. In a previous *in vitro* study, the infiltration of macrophage inflammatory cells in and around CCM lesions was reported, especially under the reaction of acute hemorrhage (74). In mouse models, macrophages were also proven in the CCM lesions by histological analysis (75). Similarly, comprehensive transcriptome analysis also provided strong support for the evidence of immune microenvironment in CCM pathology. Through the analysis of the immune microenvironment, it was demonstrated that nearly 30% of the total cell number were accounted by immune cells, and macrophages, NK cells, monocytes, as well as B cells and T cells were all included (76–78). Microglia, as resident immune cells of the central nervous system, have also attracted much attention (79). The microglia activation within the perilesional tissue were thought to mediate hematoma resolution (80, 81). Meanwhile, microglia are also thought to be involved in the pathological processes associated with epilepsy (82, 83).

The other thing that could also link CCM to the immune response is inflammatory susceptibility. Research in animal models have revealed that the anti-inflammatory threshold was decreased when the patient suffered CCM diseases (84). Thus, there is reason to believe that an enhanced inflammatory response could also be presented in CCM individuals. Or to put it another way, patients with CCMs have an increased susceptibility to inflammation. As low fluid shear stress could be one of the characteristics of CCM lesions, it is reasonable to consider whether these conditions could contribute to the increased susceptibility to inflammation and immune responses (85). Evidence from clinical blood samples is also suggestive. In a clinical study, it was suggested that inflammatory factors in plasma samples may be associated with clinical symptoms, such as epilepsy, a common hospital admitted complaint (86).

Taken together, all these results suggested an intrinsic immune response in CCMs. In addition, modifiers associated with genetic and epigenetic aspects determined inter-individual differences in susceptibility to stressful conditions. More detailed mechanisms should be further characterized, and potential therapeutic targets remain to be identified.

## Immune and Inflammatory Mechanisms for Complications

Recurrent microbleeds, as we described previously, may be one of the most common complications of CCM (11, 12, 16, 87–90). Although the potential roles of the immune response in this disease have not been illustrated clearly, the inflammatory microenvironment following hemorrhagic events has been noted (20, 58, 91). The thrombosis and leaky blood-brain barrier would lead to the formation of a unique antigenic milieu for CCM lesions. On the one hand, it may regulate angiogenesis, leading to further progression of lesions. On the other hand, the risk of rebleeding would be further increased. Evidence from clinical samples and neuroimages defined the immune microenvironment in the characteristic vascular phenotype (58, 92, 93). Clearly, repeated hemorrhagic events could be partially regarded as the ‘trigger’ of the immune response. The defected blood brain barrier might be responsible for repeated leakages, and segregated caverns at various tissue stages were accumulated with chronic blood deposition and degradation products (94). The typical pathologic structures were filled with immune and inflammatory factors. Consequently, the prognosis, whether benign or malignant, is the result of immunological struggle.

The relationship between hemorrhagic events and inflammatory responses has been well described in multiple cerebrovascular anomalies, including aneurysm (95, 96), cerebral arteriovenous malformation (97), cerebral amyloid angiopathy (98, 99), and ischemic cerebrovascular disorders (100, 101). Notably, acute brain hemorrhages may not have an opportunity to lead a similar milieu with the longstanding deposition of blood products, because it would be absorbed over a normal length of time. The recurrent and chronic bleeding might be a unique characterization of the CCM lesion, and it is the long-term inflammatory cytokines that may cause further damage to the blood-brain barrier. Anti-inflammatory treatments seem to be working, by reducing bleed rate and severity. However, it is not easy to know whether the inflammation is prior to a hemorrhagic event or if the inflammatory response is resulted from bleeding. Interestingly, it is clear that innate inflammatory response could be presented with CCM, and inflammatory cells could also be found accumulating around the lesion. Thus, similar attempts of local immunologic manipulation could be encouraged to explore the novel therapeutic approaches for CCMs.

The individual who suffered CCMs was also predisposed to a lifetime risk of epilepsy. Even a single seizure may greatly decrease health-related life quality (102, 103). The hemosiderin deposition has been considered as one of the pathological backgrounds. The lesion size and location, as well as supratentorial mass effects, may be considered as risk factors. However, for whatever reason, the existence of the immune microenvironment is underpinned, and seizures are undoubtedly the result of the BBB impairment, molecule accumulation, and neuronal hyperexcitability (104). A very strong causal effect has been reported in BBB damage and ictal activities’ generating and sustaining. Distinct mechanisms, involving disequilibrium of ionic concentrations (105–109), accumulation of serum proteins (110–112), and metabolic mismatch of amino acids (113–115), have been confirmed to induce neuronal hyperexcitability during BBB dysfunction. In turn, seizures may further exacerbate this vicious cycle of pathological cascades. For example, given ISF (interstitial fluid) formation and movement would be interfered with by damaged BBB during seizures, the expulsion of waste substances that affect neuronal electrophysiological activity would be impacted (116–118). Therefore, it is plausible to assume that the characteristic immune microenvironment of CCM may be the pathological background of epileptic seizures, and repeated or persistent seizures can worsen this milieu.

Some studies are even ahead of their time. Evidence from inflammatory plasma biomarkers, which reflected the seizures and hemorrhagic activities, were gradually reported (86, 119). These results predictively implied the association between inflammatory cytokines and subsequent clinical activities. Meanwhile, several drugs were clinically tested *via* a multivariate network (120–122). Although there are still hurdles to overcome, these preclinical discoveries and biomarker validations may contribute to future therapeutic targets’ exploration.

## Prospects and Hypothesis for Future Research

Benefited from the convergence of multiple scientific and medical advances, so much progress has been made in the CCM research field in the past two decades. Germ line mutations and/or somatic mutations are the causative factors, which played major roles in endothelial homeostasis disequilibrium events. However, the whole pathogenesis is a continuum involving lesion development and growth. On the macro level, our bodies are constantly exposed to an antigenic environment. On the micro, CCM lesions also exist in the immune microenvironment (**Figure 1**). Numerous factors involving the microbiome could affect the gut-brain axis immune system, which would affect the overall immune state of the body, diversifying the severity of the disease. Meanwhile, polymorphic gene hubs, especially about the inflammation-related genes, contributed to CCM development and progression during the distinct phase. For CCM lesions, acute and chronic bleeding are both the results and the beginnings of a new vicious cycle in the context of the immune microenvironment. Moreover, the CCM pathological context itself is an immune response site. All the molecular activities at the micro-immunological levels are closely related to clinical outcomes.

To sum up, our work emphasized the importance of immune factors as genetic modifiers, providing insights into the search for new potential therapeutic targets. Strategies preventing detrimental immune responses and enhancing beneficial immune responses are being tested, and these efforts are highly expected to make targeted and efficient clinical transformation.

## Author Contributions

TT and ZP contributed equally to this work and should be considered to be co-first authors. TT reviewed related literatures and drafted the paper. ZP reviewed related literatures and drew the illustration figures. JR discussed important points and made important revisions to the paper. HZ reviewed the final version and approved the paper for publication. All authors contributed to the article and approved the submitted version.

## Funding

National Natural Science Foundation of China Award Number: 81971113

## Conflict of Interest

The authors declare that the research was conducted in the absence of any commercial or financial relationships that could be construed as a potential conflict of interest.

## Publisher’s Note

All claims expressed in this article are solely those of the authors and do not necessarily represent those of their affiliated organizations, or those of the publisher, the editors and the reviewers. Any product that may be evaluated in this article, or claim that may be made by its manufacturer, is not guaranteed or endorsed by the publisher.
